# Carbohydrate use and reduction in number of balance beam falls: implications for mental and physical fatigue

**DOI:** 10.1186/1550-2783-10-32

**Published:** 2013-07-22

**Authors:** Helena Angélica Pereira Batatinha, Carlos Eduardo da Costa, Elias de França, Igor Roberto Dias, Ana Paula Xavier Ladeira, Bruno Rodrigues, Fabio Santos de Lira, Sonia Cavalcante Correia, Érico Chagas Caperuto

**Affiliations:** 1 Mackenzie Presbyterian University, 546, Taquari St, Moóca, Sao Paulo, Brazil; 2 São Judas Tadeu University, Sao Paulo, Brazil; 3 Department of Physical Education, São Paulo State University, UNESP, SP, Brazil

**Keywords:** Maltodextrin supplementation, Artistic gymnastics, Mental fatigue

## Abstract

**Background:**

Artistic Gymnastics is a sport where athletes are frequently fatigued. One element that might influence this aspect is carbohydrate, an important energy substrate for the muscles and the CNS. Our goal was to investigate the influence of fatigue over artistic gymnastics athlete’s performance and the effects of a carbohydrate supplementation on their performance.

**Methods:**

We evaluated 15 athletes divided in 2 groups (control and fatigue) from 12 to 14 years old in two different experimental days. On the first day (water day), they did 5 sets of exercises on the balance beam (experimental protocol) ingesting only water, CG (control group) warmed up before the experimental protocol and FG (fatigue group) did a fatigue circuit, warm up exercises and then the experimental protocol. On the second day (carbohydrate day), we used the same protocol but CG ingested a sugar free flavored juice and FG ingested a 20% concentration maltodextrin solution before the protocol on the balance beam.

**Results:**

We observed a greater number of falls from the balance beam from the FG on the first day (5.40 ± 1.14 FG vs 3.33 ± 1.37 CG; p = 0.024) and a decrease in the number of falls on the second day (2.29 ± 1.25 FG water day vs 5.40 ± 1.14 FG carbohydrate day; p = 0.0013). Carbohydrate solution was able to supply muscle demands and improve the athlete’s focus showed by the reduced number of falls.

## Background

Artistic Gymnastics training submits athletes to the limit of their bodies and minds through hard training sessions and a competitive schedule that is long and demanding both physically and mentally. Often, athletes train in a state of fatigue and close to their limits. Muscular fatigue is a process that impairs performance, especially with the athlete under caloric restriction, a common feature of this sport modality [1].

Carbohydrate supplementation may be a strategy to counteract this process, since carbohydrate is an important source of energy to the body and to the nervous system, improving the athlete performance [2]. The question that bred this study then was: what is the influence of fatigue on the athlete performance in an exercise that is highly demanding both, physically and mentally, such as the balance beam? And what would be the role of carbohydrate supplementation in this process?

Artistic gymnastics involves physical strength, concentration and gracefulness. The athletes are submitted to the limit of their bodies, there is an intense overload which requires a lot of effort from the athlete [3,4].

The balance beam is the more technical apparatus because it’s a 10 cm wide surface set at 125 cm high and the athletes must perform all movements on it and without falls [5]. The best result is obtained by the athlete who executes determined movements in its perfect form and don’t fall. Any imperfect movement caused, for instance, by fatigue, can make the athlete fall.

Being an individual sports, where all eyes are focused on the athlete at the time of the presentation no errors are accepted, the perfect execution and performance are highly valued [6]. Training is usually exhaustive, both long and of high intensity. Young athletes train an average of 25 hours per week, divided in 5 sessions of 5 hours each [4]. The competition schedule is all year long [7] therefore periodization of the training sessions is not well established. It is mostly based on a large training volume and a very high intensity, keeping the athletes close to their top performance and their limits during all the training period.

A gymnast diet is restricted to few calories [8], based on the idea that the lighter the body, less energy is needed to perform the exercises and more gracefully the athlete will do the movements. Also, the risk of injuries decreases, because the impact on the joints will be reduced.

However, with these conditions there can be a lack of energy for the athlete to complete her set of exercises or it can impair the planned routine, bringing back the injury risk [9,10].

The use of sports supplements by gymnastics athletes is very rare, being caloric restriction the main nutritional strategy for this population.

Carbohydrates supplements might be useful, since is well established as an ergogenic resource [11], being considered an essential energy supply for high intensity exercise [12] an immediate energy source either to the muscle tissue or to the nervous system, as a critical fuel for neurons [13], delaying fatigue that might be seen as an interruption of the information traffic from the brain to the muscle [14].

Therefore, the aim of this study was to investigate the influence of fatigue on the artistic gymnastic athlete performance and the influence of carbohydrate supplementation on their performance and fatigue.

## Methods

### Sample and ethical aspects

15 female artistic gymnastic athletes, from 11 to 14 years old, took part in the study. All of them were healthy and had a high training level, at least 5 times a week, 4 hours a day. Athletes were selected from the kids Barueri training team and they had at least 2 years of experience.

The study design was submitted to the Ethical Committee of Mackenzie Presbyterian University, and was in accordance with the Helsinki Declaration (1975). After the approval (under the protocol number CAAE 0032.0.272.000-10), because the subjects were under 18, we set up a meeting with the athletes coach and their parents, so they could be informed of the study procedures and sign an informed consent form if they agreed with the study. During the study, subjects were taught to leave the study protocol if they wanted or felt any discomfort.

### Experimental procedures

Athletes were divided randomly in two groups, control group (CG), and the previously submitted to fatigue group (FG).

On the first day (WATER DAY) CG did a previous warm up of 10 minutes followed by 5 sets of determined exercises (Hanging straight leg raise, scale, gymnastic turns, handstands, cartwheel, Split Leaps, walkover, a dismount with front flip) on the balance beam. FG did a fatigue circuit of 20 minutes, a 10 minutes specific warm up and then the 5 sets of the same exercises of CG.

The fatigue circuit consisted of 3 sets of 10 exercises usually performed by artistic gymnastic athletes. The protocol was very intense; the athletes reported that it was close to 90% of the rate of perceived exertion. Exercises familiar to the athletes were chosen and their coach helped to keep the athletes performing them at high intensity up to the end of the 20 minutes. The objective of the fatigue circuit was to simulate a competition day, where the balance beam is the last apparatus to be performed.

On the second day of experiment (CARBOHYDRATE DAY) the same protocol was followed, although CG athletes ingested a sugar free flavored juice solution before the warm up and FG ingested a 20% maltodextrin solution (the same flavor of CG) right after the fatigue circuit and 10 minutes prior to the warm up.

### Data collection

The number of falls during the set of specified exercises was counted in order to assess the level of fatigue and its influence on their performance.

For the CG group, blood glucose (Accu-check active Roche^®^) and Lactate (Accutrend Lactate, Roche^®^)was measured on three moments–before the warm up (REST), before the beam balance set (PRE SERIES) and immediately after the set (POS SETS).

For the FG group, blood glucose and Lactate was measured during four moments–before the fatigue circuit (REST), before the warm up and after the fatigue (FATIGUE), before the beam balance set (PRE SETS), and immediately after the set (POS SETS).

### Experimental design

On both experimental days, WATER DAY and CARBOHYDRATE DAY, we counted the number of falls during the sets on the balance beam, measured blood glucose and lactate in three moments: rest, before the sets and after the sets. For the fatigue group, we also measured blood glucose and lactate right after the fatigue circuit (Table 1).

**Table 1:** Scheme of the experimental design

**Experimental days/Groups**	**CG**	**FG**
WATER DAY (DAY 1)	Rest	Rest
		20 minute fatigue
	10 min Warm up	10 min Warm up
	5 sets	5 sets
CARBOHYDRATE DAY (DAY 2)	Rest	Rest
		20 minute fatigue
	Flavored Juice	Maltodextrin
	10 min warm up	10 min warm up
	5 sets	5 sets

### Statistical analysis

We used a two way ANOVA analysis, considering fatigue and supplementation as variables, and used independent Student T test to investigate differences between the groups when observed as pairs. Results were displayed as mean ± se (mean ± standard error) and significance level was set to p < 0.05.

## Results and discussion

The glucose and lactate profile on REST was similar to both groups on both days (WATER DAY–glucose 97.0 ± 15.5 mg/dl for CG and 97.2 ± 16.7 mg/dl for FG p = 0.98). Lactate 1.6 ± 0.4 mmol/L for CG and 1.7 ± 0.3 mmol/L for FG p = 0.67); (CARBOHYDRATE DAY–glucose 94.5 ± 18.0 mg/dl for CG and 88.0 ± 8.2 mg/dl for FG p = 0.48; Lactate 1.2 ±0.4 mmol/L for CG and 1.4 ± 0.2 mmol/L for FG p = 0.19).

The fatigue protocol was efficient, showed by a significant increase on lactate and blood glucose concentration to FG on FATIGUE (after the fatigue circuit) on both days comparing to REST (WATER DAY–lactate 13.92 ± 1.48 mmol/L FATIGUE and 1.17 ± 0.42 mmol/L REST p = 0.00007 glucose 118 ± 39.07 mg/dl FATIGUE and 97.2 ± 16.72 mg/dl REST p = 0.12); (CARBOHYDRATE DAY–lactate 10.2 ± 3.0 mmol/L FATIGUE and 1.4 ± 0.2 mmol/L REST p = 0.00007 glucose 112.0 ± 11.44 mg/dl FATIGUE and 88.0 ± 8.25 mg/dl REST p = 0.0007). The increase in glucose concentration with consequent lactate production is a response to the high intensity exercise represented by the fatigue protocol, as seen in some classic studies [15-17]. The HPA axis activation in response to stress is different when the stress is promoted by a stimulus that shows no threat; it elicits a more attenuated response [18]. Although subjects were experienced athletes and the exercises used in the fatigue protocol were all familiar to them, the physical stress was strong enough to generate the response observed.

Lactate concentration decreased significantly during warm up on FG on both days (PRE SETS compared to FATIGUE). The warm up specific exercises had its own particular purpose for the athletes but it might have worked as an active recovery process regarding the metabolic response to fatigue protocol, as described by [19].

Lactate concentration was not different when compared to CG on PRE SETS (WATER DAY–lactate 3.94 ± 3.23 mmol/L FG and 2.2 ± 0.81 mmol/L CG p = 0.27) (CARBOHYDRATE DAY–lactate 5.2 ± 1.5 mmol/L FG and 4.75 ± 2.83 mmol/L CG p = 0.73) probably because of the warm up exercises that might have helped to clear the lactate. Although the FG athletes might have recovered their lactate concentration levels, they showed some visual signs of fatigue and they reported to us as feeling fatigued, although we can’t consider that as a measured variable.

Lactate did not show any differences on both points PRE SETS and POST SETS on WATER DAY (2.2 ± 0.8 mmol/L PRE SETS and 2.3 ± 1.4 mmol/L POST SETS for CG p = 0.88 and 3.94 ± 3.23 mmol/L PRE SETS and 3.68 ± 1.87 mmol/L POST SETS for FG p = 0.91), probably because exercise intensity was constant during the set. This data corroborates the hypothesis that although the balance beam is one of the most difficult exercises in gymnastics, it is not mainly physically demanding, but it also requires a lot of concentration in order to perform it properly [6].

On CARBOHYDRATE DAY, lactate concentration didn’t change on PRE SETS and POST SETS to CG but was significant lower on POST SETS when compared to PRE SETS to FG (4.75 ± 2.83 mmol/L PRE SETS and 3.30 ± 1.32 mmol/L POST SETS for CG p = 0.22; 5.2 ± 1.5 mmol/L PRE SETS and 3.7 ± 1.2 mmol/L POST SETS for FG p = 0.03). Lactate values were lower on post sets to FG as a consequence of the stronger removal that was elicited by the higher lactate concentration produced by the fatigue circuit.

**Figure 1:**
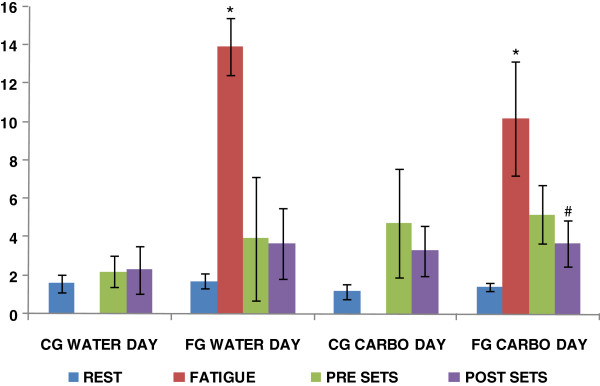
Lactate (mmol/L) data to CG and FG on both days. * p < 0.05 Comparing lactate on FATIGUE with RESTfor the FG group on both days. # p < 0.05 comparing lactate from POST SETS to PRE SETS on both days.

Lactate data can be observed on Figure 1.

On WATER DAY, glucose concentration did not change at any moment, except for the FG on FATIGUE, which showed a trend to a higher glucose concentration compared to rest (WATER DAY–97.2 ± 16.72 mg/dl FG REST; 118 ± 39.1 mg/dl FG FATIGUE p = 0.12) this glucose increase happened due to the high intensity of the fatigue protocol and the consequent hormonal responses to the stress stimulus, as promoted by the HPA axis activation [18]. A published article by Davis and Brown [1] states that exhaustive exercises increases the concentration of counter-regulatory hormones such as adrenalin, glucagon, and when the exercise in sustained for a longer period of time hGH and cortisol. These hormones contribute to preserve or increase the blood glucose concentration delaying mental fatigue.

On CARBOHYDRATE DAY the most interesting changes were registered. There was no difference between both groups on REST (94.5 ± 17.99 mg/dl CG and 88.0 ± 8.25 mg/dl FG p = 0.48) however, after FATIGUE, glucose concentration increased statistically to FG, because of the high intensity exercise and hormonal responses.

The counter-regulatory hormones can promote at the same time the release of hepatic glucose to the bloodstream and the decrease of blood glucose uptake by the muscle [20] favoring fat uptake instead, in order to ensure glucose to the brain and still provide energy to the working muscle, as described by Goodwin [21].

After carbohydrates supplementation (after REST), the glucose concentration of CG increased significantly (94.5 ± 17.99 mg/dl REST and 136.83 ± 13.79 mg/dl PRE SETS p = 0.001, after supplementation). Although this group showed a significant decrease on glucose on POST SETS (136.83 ± 13.79 mg/dl PRE SETS and 102.17 ± 14.08 mg/dl POST SETS p = 0.03) we did not observe an expected increase on lactate concentration (PRE SETS 4.75 ± 2.83 mmol/L and POST SETS 3.30 ± 1.32 mmol/L CG p = 0.22), an important and expected signal of muscular activity, especially in response to high intensity exercise. This result suggests a different share of the available glucose on PRE SETS between muscle and the central nervous system, probably with the glucose available being consumed by the CNS since the balance beam sets were advanced exercises, requiring high concentration and imposing energy demand to the tissue. A similar behavior was described by [22], when they describe muscle adaptation in an effort to oxidize fat when there is low carbohydrate availability, preserving the carbohydrates stock to tissues that depend predominantly on glucose, such as the brain. A low carbohydrate environment is associated with mental and physical fatigue as described by [23,24].

After carbohydrate supplementation (after FATIGUE) the FG presented a significant increase (88.0 ± 8.25 mg/dl REST and 112.0 ± 11.44 mg/dl after FATIGUE p = 0.007) possibly due to sympathetic nervous system activation and counter regulatory hormones influence. Glucose maintenance on PRE SETS (112.0 ± 11.44 on FATIGUE, before the warm up, after the fatigue protocol and 118.3 ± 18.85 on PRE SETS p = 0.43 after the carbohydrate supplementation), was different from the data presented on WATER DAY, when we observed a decrease (not significant (p = 0.16)) in glucose concentration between these two points. This maintenance was due the carbohydrate supplementation that provided a greater amount of glucose to the athletes when compared to WATER DAY (84.4 ± 12.22 mg/dl WATER DAY on PRE SETS and 118.3 ± 18.85 mg/dl CARBOHYDRATE DAY on PRE SETS).

On CARBOHYDRATE DAY, during the sets, we observed the same behavior for FG as to CG which was a significant decrease of glucose concentration (FG 118.3 ± 18.85 mg/dl PRE SETS and 95.5 ± 9.51 mg/dl POST SETS p = 0.04), caused by the uptake by the CNS and muscle. There was a significant decrease (only to FG) on lactate concentration comparing PRE SETS to POST SETS (5.2 ± 1.5 mmol/L PRE and 3.7 ± 1.2 mmol/L POST p = 0.03), suggesting again a different glucose sharing between the nervous and muscular systems.

**Figure 2:**
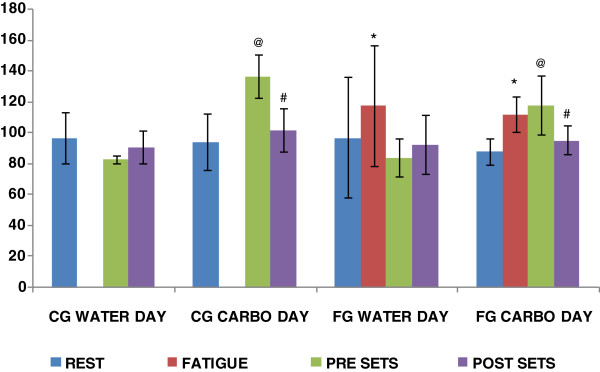
Glucose data (mg/dl) for CG and FG for both days. * p < 0.05 comparing FATIGUE to REST within the group on both days. @ p < 0.05 comparing PRE SETS to REST within the group for all groups on both days. # p < 0.05 comparing POST SETS to PRE SETS within the group for all groups on both days.

Glucose data can be observed on Figure 2.

All the metabolic results above can be corroborated by the number of falls observed during the execution of the experimental sets on the balance beam. On WATER DAY the number of falls was statistically higher to FG than CG (5.4 ± 1.14 FG and 3.33 ± 1.37 CG p = 0.02) demonstrating the effect of the fatigue protocol on the concentration status of the athletes. On CARBOHYDRATE DAY there was no difference in the number of falls between FG and CG (FG 2.29 ± 1.25 and CG 1.88 ± 1.13 p = 0.51). This lack of difference on the number of falls, might be result from the carbohydrate supplementation, which promoted a decrease in the number of falls of the FG even after the athletes did the fatigue protocol. We believe that an extra glucose supply is a fast, simple and efficient way to make a difference on muscle and mental performance [25,26].

Finally, when we compare the two different days, WATER DAY and CARBOHYDRATE DAY, we observed significant differences between the number of falls (WATER DAY CG 3.33 ± 1.37 and CARBOHYDRATE DAY CG 1.88 ± 1.13 p = 0.04) and (WATER DAY FG 5.4 ± 1.14 and CARBOHYDRATE DAY FG 2.29 ± 1.25 p = 0.01) corroborating once again the idea that the carbohydrate supplementation had a higher effect fueling the central nervous system and maintaining the glucose concentration than only as a fuel for the working muscles, although this demand has also been answered [1,22,27].

**Figure 3:**
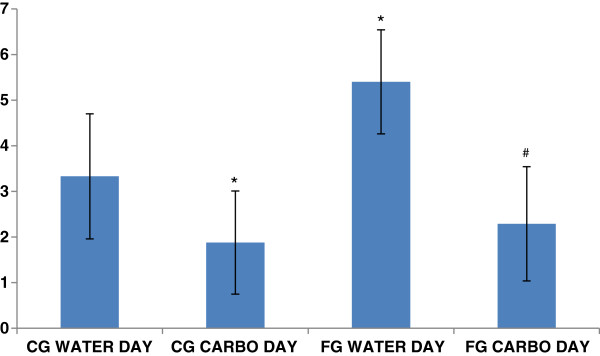
Number of falls for CG and FG on both days. *p < 0.05 compared to CG on WATER DAY. # p < 0.05 compared to FG on WATER DAY.

Number of falls data can be observed on Figure 3.

## Conclusion

We can conclude that fatigue impairs performance in artistic gymnastic athletes due to mental fatigue and consequent loss of concentration that leads to mistakes in the exercise execution.

We could also conclude that carbohydrate supplementation was able to restore the concentration levels of the athletes as well as to supply energy to the muscles, reducing mistakes or the number of falls on the balance beam, even after an exhaustive training session.

Carbohydrate supplementation can represent a legal, cheap and efficient ergogenic resource that can be the difference between a weak performance caused by fatigue or top performance, especially in a modality where caloric restriction and exhaustive training are parts of a very common routine. However, more studies are needed to the full comprehension of this phenomenon.

## Competing interests

The authors declare that they have no competing interests.

## Authors’ contributions

HAPB, CEC, EF, IRD, APXL, SCC and ECC collected data, organized and built the first drafts of the manuscript, BR and FSL joined to help improve discussion. All authors read and approved the final manuscript.
